# Effectiveness and safety of different traditional Chinese medicines for Coronavirus disease 2019: A protocol for systematic review and network meta-analysis

**DOI:** 10.1097/MD.0000000000026676

**Published:** 2021-08-06

**Authors:** Bo Huang, Yingfeng Liu, Chiheng Pi, Shifan Yan, Fusheng Li

**Affiliations:** a Jiangxi University of Traditional Chinese Medicine, Nanchang, China; b Affiliated Hospital of Jiangxi University of Traditional Chinese Medicine, Nanchang, China.

**Keywords:** COVID-19, protocol, systematic review, traditional Chinese medicine

## Abstract

**Introduction:**

The effectiveness of different Traditional Chinese Medicine in the treatment of COVID-19 is worthy of attention, but the efficacy and safety of different Traditional Chinese Medicine in the treatment of COVID-19 have not yet been compared, based on network meta-analysis.

**Methods and analysis:**

The 2 members independently searched 7 databases according to the retrieval strategy, and the retrieval time was from the beginning of the establishment of the database to June 19, 2021. Then the title was imported into the EndNote Software AQ8 (V.X9), and the duplicate literature was deleted successively, the nonconforming articles were deleted in the title reading, and finally the full text was read to determine the articles included in the study. The Cochrane Collaboration's Tool will be used to evaluate the article quality, and Stata Statistical Software (Version 14.0, Stata Corporation, College Station, TX) will be used for data analysis. Levels of evidence are evaluated according to the Grading of Recommendations Assessment, Development and Evaluation (GRADE) instrument.

**Results:**

The efficacy and safety of different Traditional Chinese Medicine in the treatment of COVID-19 were evaluated, and the order was determined according to the value of sucre.

**Conclusion:**

This study will provide evidence for the treatment of COVID-19 with TCM therapy, and provide ideas for the clinical treatment of COVID-19.

**INPLASY registration number:**

No. INPLASY202160092.

## 1 Introduction

In 2019, there was an outbreak of pneumonia of unknown cause in Wuhan, China. The main symptoms are fever, dry cough, and fatigue. It is highly contagious and has a high mortality rate.^[1,2]^ After a series of examinations, it was finally found to be caused by severe acute respiratory syndrome Coronavirus 2 (SARS-CoV-2). It was named Coronavirus Disease 2019 (COVID-19) by the World Health Organization on February 11, 2020.^[3]^ Until June 22, 2021, a total of 179.41 million people in more than 200 countries have been infected with or are being infected with the new coronavirus vaccine. Although the vaccine has been developed, the epidemic is still serious. The epidemic in China has been brought under full control, and there are still 11.47 million people abroad who have not recovered. On the other hand, the discovery of new mutant strains is also constantly threatening the safety of human beings.

The epidemic in China has been brought under control in a short time, and traditional Chinese medicine has played a big role. COVID-19 belongs to the category of “plague” in traditional Chinese medicine.^[4]^ Chinese medicine is recommended to treat COVID-19 in the third and fourth editions of COVID-19 diagnosis and treatment guidelines issued by the Chinese Health Commission,^[5,6]^ which has been proved to have certain clinical efficacy. Although some scholars conducted related studies^[7]^ in the early stage, there has been no study on reticulomete analysis, and new high-quality RCTs have been published successively. Meanwhile, in order to further verify the efficacy and safety of traditional Chinese medicine in the treatment of COVID-19, we will conduct this study.

## 2 Methods

### 2.1 Study registration

We have registered at https://inplasy.com/ and obtained the registration number No. Inplasy82060849. We will strictly perform this protocol by following the Preferred Reporting Items for Systematic Reviews and Meta-Analyses Protocol (PRISMA-P) statement guidelines.

### 2.2 Inclusion criteria for study selection

#### 2.2.1 Type of studies

All randomized controlled trials using traditional Chinese medicine to treat COVID-19 will be accepted. No language or publication status requirements. In addition, relevant nonrandomized controls, reviews, individual cases, etc., were excluded.

#### 2.2.2 Types of participants

All randomized controlled trials using traditional Chinese medicine to treat COVID-19 will be accepted. No language or publication status requirements. In addition, relevant nonrandomized controls, reviews, individual cases, etc., were excluded.

##### 2.2.2.1 Experimental interventions

The intervention measures of the experimental group were the treatment of COVID-19 with traditional Chinese medicine combined with or without western medicine, and the dose and frequency were not limited.

##### 2.2.2.2 Control interventions

The treatment of the control group could be TCM combined with Western medicine or Western medicine alone.

#### 2.2.3 Types of outcome measures

##### 2.2.3.1 Primary outcomes

1. Cure rate.
2. Aggravation rate.
3. Mortality rate

##### 2.2.3.2 Additional outcomes

1. The negative conversion rate of nucleic acid test for SARS-CoV-19
2. Any adverse events.

### 2.3 Search methods

We will draw from 7 databases: PubMed, Cochrane Central Register of Controlled Trials, EMBASE, Web of Science, Chinese Biomedical Literature Database, Wanfang Database, The Chongqing VIP Database, and Chinese National Knowledge Infrastructure were used to retrieve the randomized controlled studies on the treatment of 2019 Novel coronavirus pneumonia with Chinese medicine. The time limit was from the establishment of the Database to June 19, 2021. Meanwhile, we will also look for trials that have not yet been published in The ClinicalTrials.gov, Chinese Clinical Trial Registry. The specific PubMed retrieval strategy is shown in Table 1.

**Table 1:** Search strategy used in PubMed database.

Number	Search items
#1	randomized controlled trial [pt]
#2	controlled clinical trial [pt]
#3	randomized [tiab]
#4	clinical trials as topic [mesh: noexp]
#5	randomly [tiab]
#6	trial [ti]
#7	OR/ #1–#6
#8	COVID-19 [Mesh]
#9	COVID 19 OR COVID-19 Virus Disease OR COVID 19 Virus Disease OR COVID-19 Virus Diseases OR Disease, COVID-19 Virus OR Virus Disease, COVID-19 OR COVID-19 Virus Infection OR COVID 19 Virus Infection OR COVID-19 Virus Infections OR Infection, COVID-19 Virus OR Virus Infection, COVID-19 OR 2019-nCoV Infection OR 2019 nCoV Infection OR 2019-nCoV Infections OR Infection, 2019-nCoV OR Coronavirus Disease-19 OR Coronavirus Disease 19 OR 2019 Novel Coronavirus Disease OR 2019 Novel Coronavirus Infection OR 2019-nCoV Disease OR 2019 nCoV Disease OR 2019-nCoV Diseases OR Disease, 2019-nCoV OR COVID19 OR Coronavirus Disease 2019 OR Disease 2019, Coronavirus OR SARS Coronavirus 2 Infection OR SARS-CoV-2 Infection OR Infection, SARS-CoV-2 OR SARS CoV 2 Infection OR SARS-CoV-2 Infections OR COVID-19 Pandemic OR COVID 19 Pandemic OR COVID-19 Pandemics OR Pandemic, COVID-19 [All Fields)
#10	OR/#8–#9
#11	Medicine, Chinese Traditional [Mesh]
#12	Traditional Chinese Medicine OR Traditional Medicine, Chinese OR Chinese Traditional Medicine OR Chinese Medicine, Traditional [All Fields)
#13	#11OR #13
#14	7 AND #10 AND #13

### 2.4 Data collection and analysis

#### 2.4.1 Selection of studies

**Figure 1:**
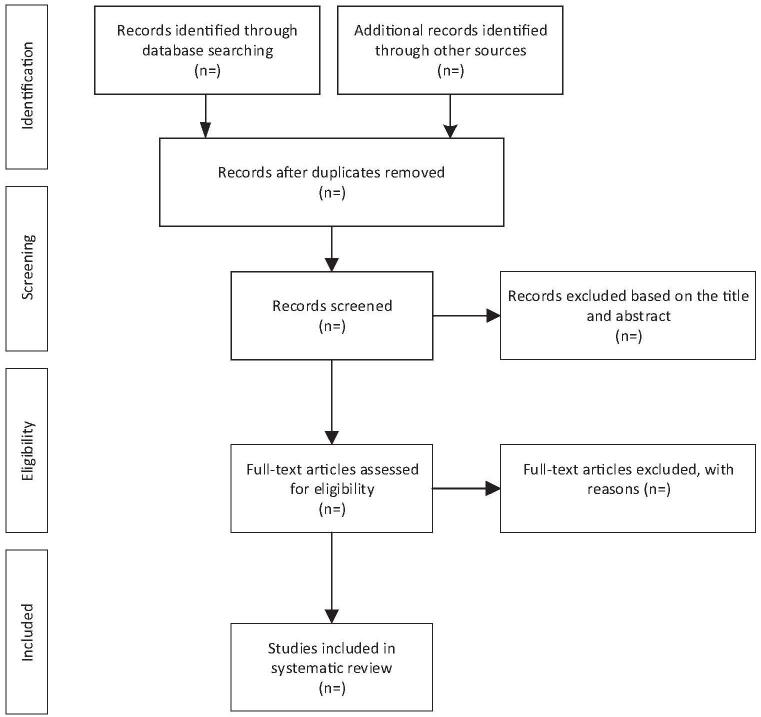
Flow diagram of study selection process.

All the literatures were imported into Endnote software (v.X9) to delete the duplicates. Two team members (BH and YL) independently read the biblios, deleted the articles that did not meet the criteria for inclusion in the study, and further read the full text to select the articles that were finally included in the study. Then 2 team members (BH and YL) will exchange the results and check them. If there is any disagreement, it will be decided by the group discussion. The specific literature screening flow chart is shown in Figure 1.

#### 2.4.2 Data extraction and management

Two team members (BH and YL) will independently extract the following information in the article and input it into the data extraction information table prepared in advance

1. Article information: publication year, journal, author.
2. Basic information of participants: gender, age, course of disease, country, and sample size.
3. Research methods: randomization, allocation concealment, blind method, result analysis.
4. Intervention: drug, dose, frequency, and course of treatment.
5. Outcome measurement: primary and secondary outcomes.

### 2.5 Evaluation of bias risk in included studies Cochrane

The Cochrane Bias Risk Assessment Tool^[9]^ will be used to assess the quality of articles by 2 team members (BH and YL), and the assessment will mainly include seven aspects: random sequence generation, allocation concealment, blinding of participants and personnel, blinding of outcome assessment, incomplete outcome data, selective outcome reporting, and other bias. Each project has 3 levels, high, low, and undefined risk deviation levels. When relevant information is missing, we will email the author to obtain the original information. If the result is in dispute, the final result will be decided by the group discussion.

### 2.6 Data synthesis

Assessment of heterogeneity. Heterogeneity tests for all included studies were performed by using Network prediction interval graph, then to study the relationship of the weighted mean difference at a 95% confidence interval (95% CI) and estimation zone (95% Prl) to invalid line, only when invalid line crosses perpendicularly to estimation zone but does not to CI, then it means that heterogeneity exists. If there is a direct comparison between the experimental interventions included in the data, the Stata14.0 will be used for pairwise meta-analysis based on a random-effects model. Network meta-analysis. Two team members (BH and YL) used statistical software Stata (version 14.0; Stata Corporation, College Station, TX) for analysis. A random effects model was used for network meta-analysis to compare the variables between different interventions. By comparing Surface Under the Cumulative Ranking Curve (SUCRA), the optimum intervention measures were determined. The range of SUCRA is 0% to 100%; the higher of the cumulative ranking curve means the better of the efficacy.

### 2.7 Management of missing data

If the data in the included article is missing or incomplete, we will contact them via email to obtain the relevant information. If the above methods are not successful, we will use the obtained data for analysis. If the analysis is not allowed, relevant articles will be excluded.

### 2.8 Subgroup analysis

When there is obvious heterogeneity in the included articles, we will conduct subgroup analysis to reduce the heterogeneity, and analyze it from the aspects of drug dose, frequency, treatment course, etc.

### 2.9 Sensitivity analysis

We will exclude low-quality articles to test whether the conclusions of the meta-analysis are credible.

### 2.10 Assessment of reporting biases

Funnel plots will be used to detect Na publication bias. If the number of included articles exceeds 10, the 2 sides of the funnel plots are asymmetrical, Egger test will be further conducted for analysis.

### 2.11 Quality of evidence

Two team members (and) will independently use the Grading of Recommendations Assessment, Development, and Evaluation (GRADE)^[10]^ to evaluate the evidence, and the Evaluation result has 4 grades: high, medium, high low, and very low levels.

### 2.12 Ethics and dissemination

This study does not involve patients’ private information and does not require ethical approval. The result of this scheme will be Circulate in peer-reviewed journals.

## Author contributions

**Data curation:** Bo Huang.

**Formal analysis:** Bo Huang.

**Investigation:** Yingfeng Liu.

**Methodology:** Yingfeng Liu, Shifan Yan, Fusheng Li.

**Project administration:** Yingfeng Liu.

**Supervision:** Chiheng Pi, Shifan Yan.

**Writing – original draft:** Fusheng Li.

**Writing – review & editing:** Chiheng Pi.
